# P2×7 purinergic signaling in dilated cardiomyopathy induced by auto-immunity against muscarinic M_2_ receptors: autoantibody levels, heart functionality and cytokine expression

**DOI:** 10.1038/srep16940

**Published:** 2015-11-23

**Authors:** Camila Guerra Martinez, Daniel Zamith-Miranda, Marcia Gracindo da Silva, Karla Consort Ribeiro, Izaíra Trincani Brandão, Celio Lopes Silva, Bruno Lourenço Diaz, Maria Bellio, Pedro Muanis Persechini, Eleonora Kurtenbach

**Affiliations:** 1 Instituto de Biofísica Carlos Chagas Filho, Universidade Federal do Rio de Janeiro, Rio de Janeiro, RJ, 21941-902, Brasil; 2 Instituto de Microbiologia Prof. Paulo de Goes, Universidade Federal do Rio de Janeiro, 21941-900 Rio de Janeiro, RJ, Brasil; 3 Departamento de Bioquímica e Imunologia, Faculdade de Medicina de Ribeirão Preto, Universidade de São Paulo, Av. Bandeirantes 3900, Ribeirão Preto, SP, Brasil; 4 Instituto Nacional de Ciência e Tecnologia para Pesquisa Translacional em Saúde e Ambiente na Região Amazônica, Conselho Nacional de Desenvolvimento Científico e Tecnológico/MCT, Rio de Janeiro, Brasil; 5 Instituto Nacional de Propriedade Industrial. Rua São Bento no 1, Rio de Janeiro, RJ, 20090-010, Brazil

## Abstract

Autoantibodies against the M_2_ receptors (M_2_AChR) have been associated with Dilated Cardiomyopathy (DCM). In the heart, P2×7 receptors influence electrical conduction, coronary circulation and response to ischemia. They can also trigger pro-inflammatory responses and the development of neurological, cardiac and renal disorders. Here, P2×7^−/−^ mice displayed an increased heart rate and ST segment depression, but similar exercise performance when compared to wild type (WT) animals. After immunization with plasmid containing M_2_AChR cDNA sequence, WT mice produced anti-M_2_AChR antibodies, while P2×7^−/−^ mice showed an attenuated production. Despite this, WT and P2×7^−/−^ showed left ventricle cavity enlargement and decreased exercise tolerance. Transfer of serum from M_2_AChR WT immunized mice to näive recipients led to an alteration in heart shape. P2×7^−/−^ mice displayed a significant increase in the frequency of spleen regulatory T cells population, which is mainly composed by the FoxP3^+^CD25^−^ subset. M_2_AChR WT immunized mice showed an increase in IL-1β, IFNγ and IL-17 levels in the heart, while P2×7^−/−^ group produced lower amounts of IL-1β and IL-17 and higher amounts of IFNγ. These results pointed to previously unnoticed roles of P2×7 in cardiovascular and immune systems, and underscored the participation of IL-17 and IFNγ in the progress of autoimmune DCM.

Dilated cardiomyopathy (DCM) is the most common form of heart muscle disease, accounting for 60% of cardiomiopathies. It is primarily characterized by an enlargement of the heart cavities, particularly the left ventricle. Systolic contractile dysfunction and a reduction of up to 50% in the ejection fraction are also observed. DCM can be the final outcome of several diseases and/or injuries, including ischemia, hypertension, alcohol toxicity, previous viral and non-viral infections, genetic abnormalities and autoimmune disorders^1^,^2^.

In the 90’s, several authors described that patients with DCM presented antibodies against muscarinic acetylcholine receptor subtype M_2_ (M_2_AChR), with the second extracellular loop (M_2_AChR-el_2_) the most characterized epitope^3^. These antibodies reproduce the negative chronotropic effect of muscarinic agonists in isolated heart, an effect inhibited by the muscarinic antagonist atropine^4^,^5^. Antibodies against M_2_AChR-el_2_ induced a higher decrease in the heart rate of mice treated with carbachol, indicating that anti-M_2_AChR antibodies lead to a reduction of parasympathetic tone^6^. In addition, a study with 104 DCM patients demonstrated that those positive for anti-M_2_AChR (40%) displayed a higher incidence of atrial fibrillation when compared with anti-M_2_AChR-negative patients^7^.

Besides auto-antibodies, several other immune mechanisms are important in the pathophysiology of the mammalian heart^8^. A series of experiments have established the involvement of IFNγ and IL-17 in autoimmune disorders and their severity^9^,^10^. These cytokines are produced mainly by Th1 and Th17 cells, respectively^11^, although in some instances it is also possible to observe a shift from Th17 cells to a Th1 phenotype, where Th17 cells can synthesize IFNγ^12^,^13^. The role of these two cytokines has been extensively studied in DCM models^14^,^15^,^16^ and their importance seems to be dependent on the study model used^11^.

In experimental autoimmune myocarditis model (EAM) using myocardiotogenic peptide derived from alpha-cardiac myosin heavy chain accompanied for 62 days, IFNγ presented a protective role. This finding was well reported by using *Ifng*^−/−^ mice that showed an increased in heart dilatation and Left Ventricle (LV) mass increment around the 23^th^ day^15^,^16^,^17^. In an opposite way, using IL17 deficient mice, these authors showed that this cytokine played a harmful role especially promoting heart remodeling, fibrosis and dilatation^15^,^16^,^17^.

Indeed, in the cases where the progression of DCM takes a longer time, such as in animals or patients with Chagas’ disease that has evolved into the cardiac form, IFNγ has been classically characterized as a key injurious element^18^,^19^. Also, it has been shown recently that the amount of IL-17 is an important factor in this situation^20^,^21^. Chagas’ patients showing ejection fraction around 35% had lower levels of CD3^+^CD4^+^IL17^+^ cells and higher IFNγ compared with those with preserved global left ventricular function (higher than 50%). In overall, these results suggest that the balance of these two cytokines may influence the evolution of dilated cardiac pathogenesis.

Our group established a model where mice immunized with the pcDNA3-hM_2_, a DNA plasmid carrying the entire M_2_AChR cDNA sequence, developed anti-M_2_AChR–associated DCM that mimicked important characteristics of human DCM, such as a decrease in LV wall thickness and a reduction in fractional shortening (FS)^22^. Also, the histopathological analysis of their hearts revealed disarray in the organization of myofibrils and the presence of areas of fibrosis. Thus, this plasmid-based model opened new possibilities to a better understanding of immune- and non-immune mechanisms underlying the development of DCM.

Particularly, P2×7 receptors are important modulators of the immune response which participate in the activation of NALP3 inflammasome. This inflammasome assembly requires the presence of danger molecules such as urate crystals, viral RNA/DNA and the bacterial-derived lipopolysaccharide LPS and extracellular ATP stimulus resulting in a reduction of intracellular K^+^ concentrations^23^. As consequence the caspase 1 activation and the processing and release of mature IL-1β and IL-18 by macrophages and other cell types occur, triggering pro-inflammatory immune responses and pyroptotic cell death^23^,^24^,^25^.

Besides to be involved in antigen processing, regulatory T (Treg) cell function, leukocyte migration, and in the interaction between the innate and acquired immune systems^26^,^27^,^28^ the participation of P2×7 in cardiac system was recently described. The treatment of rodents with P2×7 antagonists improved heart functional parameters in injury/reperfusion and experimental autoimmune myocarditis models^29^,^30^,^31^,^32^, although it has also been described that exposure to extracellular ATP at preconditioning or postconditioning periods can act as cardioprotectant in acute myocardial infarction^31^.

Due to the importance of P2×7 receptors to both the cardio-vascular and immune systems, we hypothesized that they may be important players in the cascade of events that connects the administration of pcDNA3-hM_2_ DNA plasmids, the production of auto-antibodies, cytokine production, Treg cell numbers, and the development of autoimmune DCM. In the present study, C57BL6/J, WT and P2×7,^−/−^ mice were immunized with the pcDNA3-hM_2_ DNA plasmid and immunological and cardiac parameters were followed over 40 weeks post-immunization. The results allowed us to gain new insights into the role of the immune system and P2×7 receptors in the development of DCM, and to uncover the important role played by P2×7 receptors in the heart’s electrical function and in the chain of events that leads to the production of antibodies after plasmidial immunization.

## Results

### Anti-M_2_AChR serum antibody production in immunized mice

**Figure 1:**
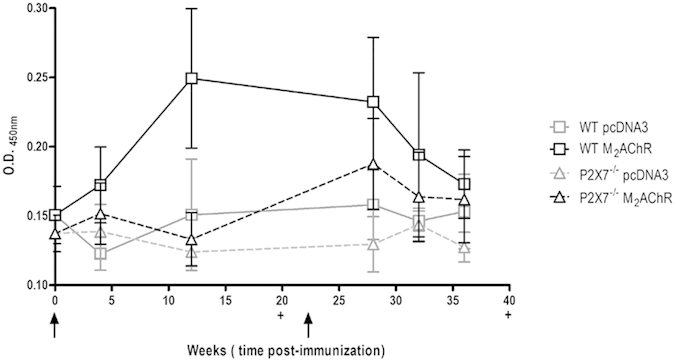
Anti-M_2_AChR antibodies production in immunized animals. The dosages of anti-M_2_AChR-el_2_ IgG antibodies produced by pcDNA3 controls (grey symbols) and pcDNA3-hM_2_ (black symbols) immunized mice were performed by ELISA. WT animals are represented by full lines and P2×7^−/−^ animals are represented by dashed lines. The values were expressed as the optic density at 450 nm. All analyses were performed in pools of serum from 10 animals. The arrows indicate time of immunization. The data points are presented as the means ± S.E.M from three independent experiments.

A synthetic peptide corresponding to M_2_AChR-el_2_ was used to detect the anti-M_2_AChR antibody production in animals immunized with the plasmid encoding the full sequence of M_2_AChR (pcDNA3-hM_2_). M_2_AChR wild type (WT – full black lines) mice produced higher levels of anti-M_2_AChR antibodies from the 5^th^ week post-immunization as compared to the control animals immunized with the empty pcDNA3 plasmid (Fig. 1 – full grey lines). The levels of IgG against the muscarinic receptor peaked at the 12^th^ week post-immunization and were maintained throughout the experimental protocol, with a tendency to decrease after the 28^th^ week post-immunization.

In contrast, P2×7^−/−^ mice immunized with the same plasmids (Fig. 1 – dashed black lines) presented a different antibody profile, with initially lower levels of anti-M_2_AChR antibodies that increased only at the 28^th^ week post-immunization, four weeks after the second set of immunizations. Control P2×7^−/−^ animals immunized with the empty pcDNA3 plasmid (Fig. 1 – dashed grey lines) did not produce significant amounts of anti- M_2_AChR antibodies.

### Ergometry

Next, we performed functional and heart morphological analyses to investigate comparatively the establishment of DCM in WT and P2×7^−/−^ mice.

**Figure 2:**
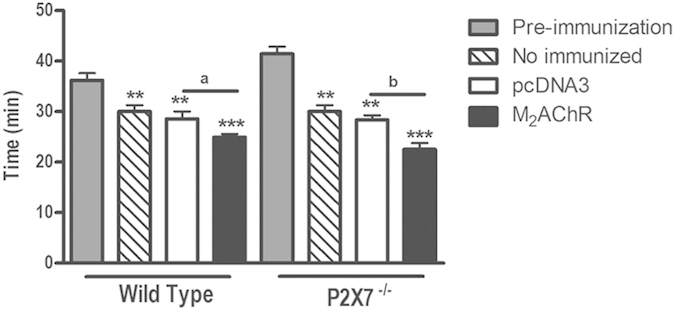
Ergometry. Mice were subjected to exercise tests on a treadmill before and 40^th^ week after plasmid immunization. The pre-immunization mice (eight weeks old) are represented by gray bars (N = 10). Non-immunized mice (N = 5, dashed bars) with the same age as immunized mice (57 weeks old) were submitted to the same test. Mice immunized with pcDNA3-hM2 are represented by black bars (N = 5) and with pcDNA3 plasmid by white bars (N = 5). The letter **a** indicates a comparison between the WT pcDNA3 and WT M_2_AChR groups, while letter **b** indicates a comparison between the P2×7^−/−^ pcDNA3 and P2×7^−/−^ M_2_AChR groups. In these cases p < 0.05 was considered statistically significant. **(p < 0.01) and ***(p < 0.001) indicate comparison of the results obtained for immunized mice groups at 40 weeks with those obtained at the pre-test (grey bar).

Using the treadmill stress test, the exercise times and maximum speeds achieved were evaluated before immunization (corresponding to animal age of eight weeks) and 40 weeks after immunization (corresponding to 57 –week old) (Fig. 2). At the 40^th^ week post-immunization, a decrease in the exercise time was observed in WT and P2×7^−/−^ mice either immunized with pcDNA3 (Fig. 2 - white bars) or M_2_AChR (Fig.2 - black bars) and also in non-immunized mice (Fig. 2 - dashed bars) as compared with pre-immunization animals tests (Fig. 2 - grey bars). Notably, mice that did not receive any plasmid immunizations displayed average exercise times (Fig. 2 - dashed bars) equal to those of age-matched mice immunized with the empty plasmid pcDNA3 (Fig. 2 - white bars). So this decrease might reflect the aging of the mice.

In addition, WT and P2×7^−/−^ animals immunized with pcDNA3-hM_2_ (Fig. 2 - black bars) displayed a significant reduction in the exercise times when compared with animals immunized with pcDNA3 at the 40^th^ week post-immunization (Fig. 2 - white bars). This drop should be interpreted as a cardiac impairment in the M_2_AChR WT group like that which we have previously described in BALB/c animals^22^. Moreover, M_2_AChR P2×7^−/−^ mice also presented a reduction in exercise time, suggesting that these mice may also have developed a similar pathology, even in the presence of low M_2_AChR auto-antibody titers.

### Morphometric analysis

**Figure 3:**
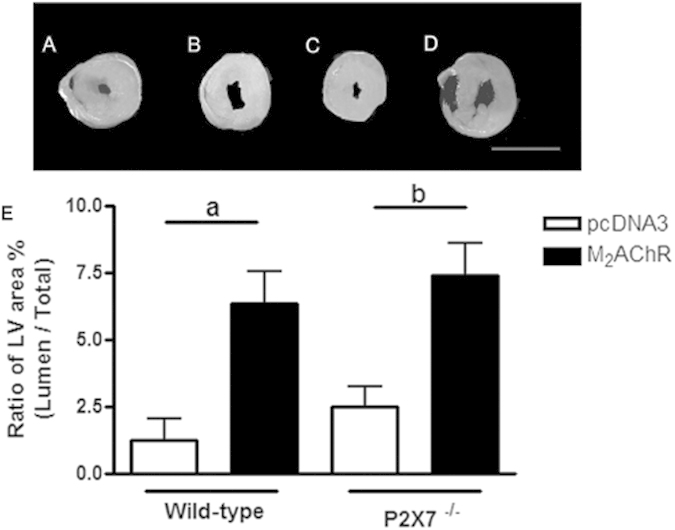
Morphometric analysis of the heart. Hearts of WT and P2×7^−/−^ mice, immunized with pcDNA3 or pcDNA3 M2AChR, were collected and analyzed at 40 weeks post-immunization. Upper panel: (**A–D**) Representative LV myocardial cross section micrographs of hearts from animals of each group at 40 weeks post-immunization. Scale bar, 2 cm. Lower panel: (**E**) Left Ventricle (LV) area ratio between the area of the cross-section of lumen and the total area of the cross-section of the heart. Black bars represent M_2_AChR groups and white bars pcDNA3 groups. The letter **a** indicates a comparison between the WT pcDNA3 and WT M_2_AChR groups, while letter **b** indicates a comparison between the P2×7^−/−^ pcDNA3 and P2×7^−/−^ M_2_AChR groups. N = 5, p < 0.05 was considered statistically significant.

The exercise intolerance previously observed could be a consequence of a commitment at cardiac function or/and morphology. To investigate this hypothesis, heart morphological analysis was performed at the 40^th^ week post-immunization. As shown in Fig. 3, heart samples from M_2_AChR WT and P2×7^−/−^ groups displayed an enlargement in the lumen internal area of left ventricle cross section (Fig. 3 - panels B and D) as compared to the pcDNA3 control groups (Fig. 3 - panels A and C).

One of the typical features of DCM is a significant left ventricle dilation and wall thinning. When this effect is observed the ratio between the lumen internal area and total area of left ventricle increases^33^. These two measurements were evaluated by using ImageJ software and as seen in the graph at Fig. 3 (lower panel). Both WT and P2×7^−/−^ animals of the M_2_AChR group (Fig. 3, black bars) showed LV area ratios (lumen/total) around three times higher than pcDNA3 control animals (Fig. 3 - white bars).

### Electrocardiographic analysis

The electrical activity of the mice was assessed by electrocardiography (ECG) during the pre-immunization period and at the 5^th^, 10^th^, 20^th^ and 40^th^ weeks post-immunization as shown in Table 1. No statistically significant RR interval differences were observed between pcDNA3 and M_2_AChR WT mice group, although the Two-way ANOVA statistic test showed an effect over the weeks (p < 0.001). Taking into account the P2×7^−/−^ animals, it was noted that the M_2_AChR P2×7^−/−^ group tended to be more bradycardic than animals belonging to the pcDNA3 P2×7^−/−^ control group. This decrease in RR interval reflected at heart rate was statistically different at the 10^th^ week post-immunization when Bonferroni’s post test was used.

Nevertheless, P2×7^−/−^ knockout mice already showed higher heart frequencies in the pre-immunization tests when compared with WT animals. This profile was maintained in the pcDNA3 P2×7^−/−^ mice over the experimental time, as seen by RR interval values frequently smaller than those presented by pcDNA3 WT mice, notably at 10^th^ week post-immunization. P2×7^−/−^ M_2_AChR mice did not keep the profile of high cardiac rates. Immunization of P2×7^−/−^ mice with pcDNA3-hM_2_ plasmid gradually led to a decrease in the heart rate of this group, reaching values close to those of WT mice groups (Table 1).

Regarding the duration of the QT interval corrected for heart rate (QTc), no differences in the WT group were observed. When this parameter was analyzed by Two-way ANOVA hypothesis statistical test comparing WT and P2×7^−/−^ groups, P2×7^−/−^ mice displayed a longer QTc interval (p < 0.05), indicating defects in the depolarization of ventricles in this group, independently of pcDNA3-hM2 immunization (Table 1).

Regardless of their immunization status, P2×7^−/−^ mice displayed a displacement of the ST interval, forming an ST depression in the electrocardiogram when compared to WT mice (p < 0.001). This alteration presented by P2×7^−/−^ mice was not accompanied by drastic change in JT interval duration (Table 1).

It is noteworthy that no arrhythmias were observed in the WT or P2×7^−/−^ mice groups. Examples of the ECG traces of one representative animal belonging to each of the four immunized groups at the 10^th^ week post-immunization can be seen in Supplementary Figure S1.

The above data showed that P2×7^−/−^ mice developed DCM-like disease regardless of the reduced production of antibodies. These data suggest that other immune-related mechanisms may be involved in triggering DCM in the absence of P2×7 receptors. It is therefore important to test whether the transfer of anti-M_2_AChR WT serum can induce the development of DCM in P2×7^−/−^ animals as they do in WT animals.

Serum containing anti-M_2_AChR antibodies from WT immunized mice, at 10^th^ week post-immunization, was transferred to naive WT and P2×7^−/−^ recipient mice. After ten weeks, recipient mice were subject to heart morphometric analysis and ECG exams.

The presence of anti-M_2_AChR antibody led to an increase in the heart size and a change in its format from an ellipsoidal to a more rounded shape in WT and P2×7^−/−^ animals when compared to their respective control groups suggesting that recipient mice may develop antibody-induced DCM (Supplementary Figure S2 A–D). Analyzing myocardial cross section micrographs it was possible to observe a tendency in the ratio of heart LV lumen area to increase in WT and P2×7^−/−^animals that received serum from WT M_2_AChR, being more prominent in animals from the WT group (Supplementary Figure S2 E).

The analysis of ECGs (Table 2) detected an increase in the cardiac frequency and JT interval of WT recipient mice, when compared to those that received serum from pcDNA3 immunized WT mice. P2×7^−/−^ mice that received anti-M_2_AChR serum also displayed lower heart rates than their respective control groups, as already seen when they were immunized with pcDNA3-hM2 plasmid, indicating that these mice were also susceptible to the effect of anti-M_2_AChR serum.

Differently to what we have observed in the above experiments, when serum from M_2_AChR-immunized P2×7^−/−^ mice, shown in Fig. 1 to have lower titers of anti-M_2_AChR, was transferred into WT or P2×7^−/−^ unimmunized mice, we did not observe DCM-like changes in either heart morphology or the ECG (Supplementary Table S1). These results indicate that autoimmune components other than anti-M_2_AChR are involved in the development of DCM in P2×7^−/−^ mice under our experimental conditions.

### Characterization of Treg spleen population

**Figure 4:**
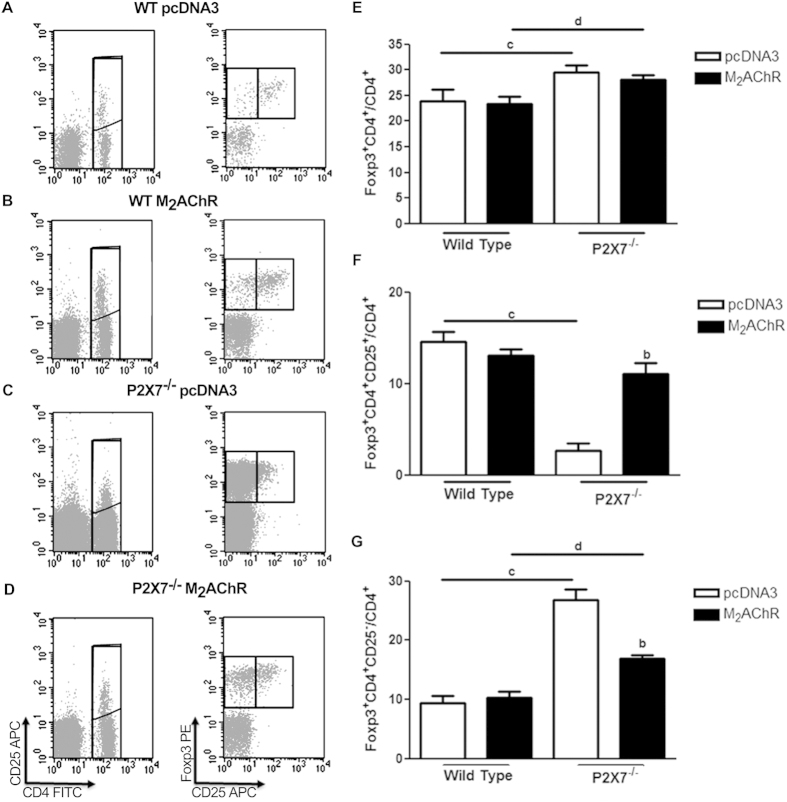
Frequency of CD4^+^ T lymphocytes and Treg in the spleen of WT and P2×7^−/−^ immunized mice. Left panel (**A–D**), representative cytometry data and gate strategy of (**A**) WT pcDNA3, (**B**) WT M_2_AChR, (**C**) P2×7^−/−^ pcDNA3 and (**D**) P2×7^−/−^ M_2_AChR individual mouse spleen cell analyzes. Mean and SD values of the frequency of (**E**) total CD4^+^ T cells; (**F**) total CD4^+^FoxP3^+^ Treg among CD4^+^ T cells and (**G**) CD4^+^FoxP3^+^CD25^−^ Treg subset among CD4^+^ T cells in each experimental group. Spleens of 5 mice in each group were individually analyzed at day 40 after immunization. Staining was performed as described in the Methods section. The letter **b** indicates a comparison between the P2×7^−/−^ pcDNA3 and P2×7^−/−^ M_2_AChR groups. Bars with **c** indicate the comparison between the WT pcDNA3 and P2×7^−/−^ pcDNA3 groups and with **d** indicate a comparison between the WT M_2_AChR and P2×7^−/−^ M_2_AChR groups. N = 5.

Regulation of immune responses to self and foreign antigens is critically dependent on Treg function. These cells are characterized by the expression of transcription factor FoxP3 in the CD4^+^ T cell population^34^. We have therefore decided to investigate the frequency of Treg cells in the spleen of pcDNA3 and M_2_AChR mice at the 40^th^ week post-immunization. Representative cytometry data of individual mouse in each experimental group and gate strategy used in our analyses are shown in Fig. 4(A–D). The total number of spleen cells (data not shown) and the percentages of total CD4^+^ T cells (Fig. 4E) showed no significant differences between the four experimental mouse groups.

In WT mice, the percentage of total Treg cells (CD4^+^FoxP3^+^), as well as the percentage of the CD25^−^ Treg subpopulation, were not significantly different in M_2_AChR group when compared to the pcDNA3 control group (Fig. 4 - F and G - 1^st^ and 2^nd^ columns).

On the other hand, in P2×7^−/−^ mice the frequency of total CD4^+^FoxP3^+^cells showed a small but significant increase when compared with their respective WT group at the 40^th^ week post-immunization (Fig. 4F – 3^rd^ column and 4^th^ column). A more detailed analysis showed that P2×7^−/−^ pcDNA3 control group had significantly higher percentage of the CD25^−^ subset of Tregs (Fig. 4G – 3^rd^ column) compared to WT controls (Fig. 4G –1^st^ column). Although the levels of this population are diminished in P2×7^−/−^ mice immunized with the plasmid coding M_2_AChR (compared to the pcDNA3 control group), the frequency of CD25^−^Foxp3^+^ Tregs is significantly higher in P2×7^−/−^ than in WT mice (Fig. 4 - G – 2^nd^ and 4^th^ columns).

These data show that although P2×7^−/−^ mice have an higher frequency of Tregs in the spleen, compared to WT mice, the majority of Tregs in pcDNA3-immunized P2×7^−/−^ mice have a CD25^−^ phenotype, which have been associated to an unstable subset of Tregs^35^.

### IL-1β production in the hearts of M_2_AChR mice is dependent on P2×7

**Figure 5:**
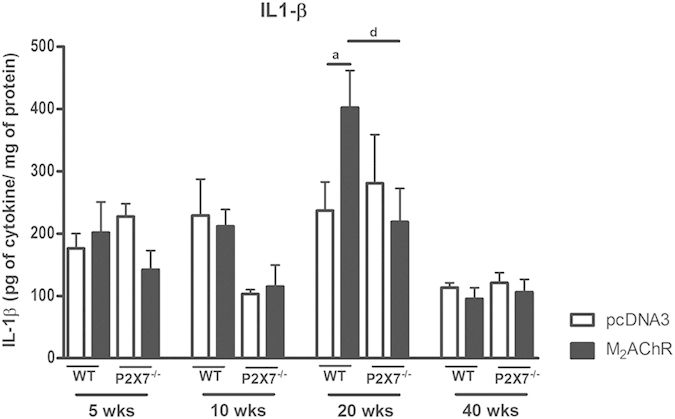
IL-1β production in the heart during 40 weeks post-immunization. The levels of IL-1β were analyzed directly in heart homogenate without any additional stimulus and expressed as pg of cytokine per mg of total protein at the 5^th^, 10^th^, 20^th^, and 40^th^ weeks post-infection. Black bars represent the M_2_AChR-immunized groups and white bars represent the pcDNA3-immunized control groups. Bar with a indicates statistically significant differences between WT pcDNA3 and WTM_2_AChR groups. The letter d indicates a comparison between the WT M_2_AChR and P2×7^−/−^ M_2_AChR groups. N = 5, p < 0.05 was considered statistically significant.

To determine the participation of the innate immune system in the establishment of the DCM, we analyzed the dosage of IL-1β in cardiac homogenates. As shown in Fig. 5, the level of IL-1β in the heart at the 20^th^ week post-immunization was higher in WT mice immunized with pcDNA3-hM_2_ plasmid as compared to pcDNA3-immunized-mice, a difference that was not observed in the hearts of P2×7^−/−^ mice. This pattern of IL-1β production is consistent with other experimental models where P2×7^−/−^ were used^36^. This profile was not maintained at the 40^th^ week post-immunization when control and immunized mice displayed low levels of IL-1β.

### IFNγ production peaks at the 20^th^ week post-immunization and is not dependent on P2×7

**Figure 6:**
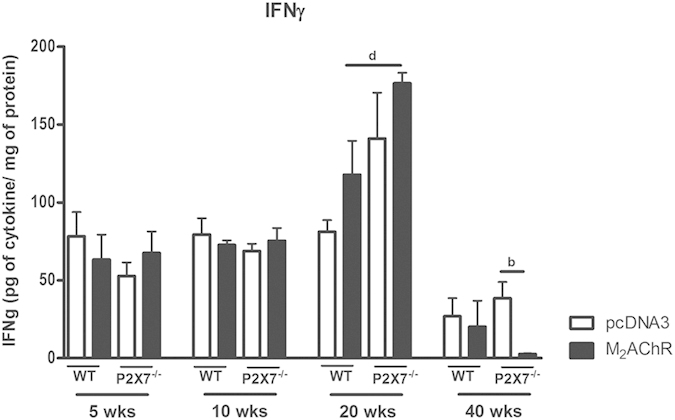
IFNg production in the heart during 40 weeks post-immunization. The levels of IFNγ were analyzed using heart homogenate samples. Black bars represent the M_2_AChR-immunized groups and white bars represent the pcDNA3-immunized control groups. Bar with b indicates statistically significant differences between P2×7^−/−^ pcDNA3 and P2×7^−/−^ M_2_AChR groups and with d indicates a comparison between the WT M_2_AChR and P2×7^−/−^ M_2_AChR groups. N = 5, p < 0.05 was considered statistically significant.

The participation of IFNγ in the cardiomyopathy induced by immunization with pcDNA3-hM_2_ plasmid was assessed through the dosage of this cytokine in heart homogenates (Fig. 6). At the 5^th^ and 10^th^ weeks post-immunization there were no significant differences amongst the four experimental groups. However at the 20^th^ week the hearts of M_2_AChR WT mice displayed an increased level of IFNγ when compared to the pcDNA3 control group and to the same group at the 5^th^ and 10^th^ weeks post-immunization. In the hearts of P2×7^−/−^ mice, an increased level in IFNγ was observed in both pcDNA3-immunized mice and, more significantly, M_2_AChR-immunized mice. At the 40^th^ week post-immunization, these values fell drastically in all groups with the P2×7^−/−^ M_2_AChR mice displaying the lowest levels of IFNγ (Fig. 6).

### IL-17 production in the hearts of M_2_AChR mice is dependent on P2×7

**Figure 7:**
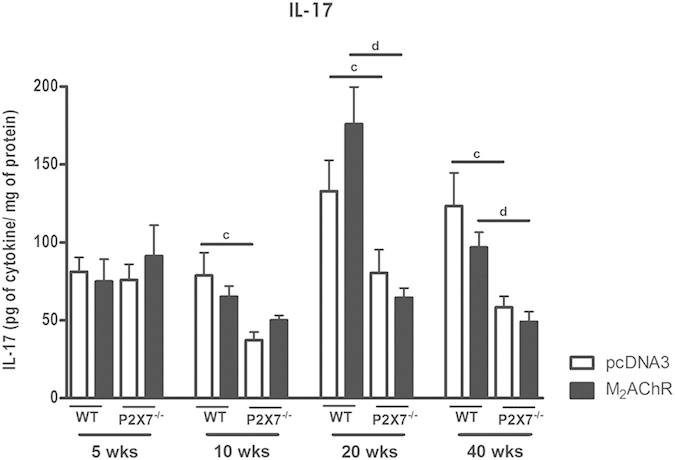
IL-17 production in the heart during 40 weeks post-immunization. The levels of IL-17 were analyzed using the same protocol as previously described for obtaining the dosage of IL-1b. Black bars represent the M_2_AChR-immunized groups and white bars represent the pcDNA3-immunized control groups. Bars with c indicate the comparison between the WT pcDNA3 and P2×7^−/−^ pcDNA3 groups and with d indicate a comparison between the WT M_2_AChR and P2×7^−/−^ M_2_AChR groups. N = 5, p < 0.05 was considered statistically significant.

We also analyzed the expression of IL-17 in the hearts during the period from the 5^th^ to the 40^th^ week post-immunization (Fig. 7). Starting at the 10^th^ week post-immunization animals from the P2×7^−/−^ groups presented lower levels of IL-17 when compared to WT groups. At the 20^th^ week post-immunization the highest IL-17 levels were detected in the hearts of M_2_AChR-immunized WT mice. Although no statistically significant difference in IL-17 levels existed between M_2_AChR-immunized and pcDNA3-immunized WT groups it seemed slightly higher in M_2_AChR WT mice (p = 0.06). IL-17 levels decreased at the 40^th^ week in all groups but both P2×7^−/−^ immunized groups showed significantly lower levels than their corresponding WT counterparts. It is noteworthy that IL-17 and IL-1β production in the heart followed the same profile, suggesting an association between these two cytokines in the progression of DCM under our experimental conditions.

## Discussion

Here we first demonstrated that C57BL/6J WT mice immunized with the M_2_AChR sequence developed similar DCM characteristics as previously described for BALB/c mice^22^. Additionally, the employment of P2×7^−/−^ mice allowed us to gain insight not only into the immune mechanism of DCM development, but also the involvement of the P2×7 receptor in cardiac function. M_2_AChR-immunized P2×7^−/−^ mice only produced anti-M_2_AChR-el_2_ antibodies at a later time and at a more attenuated level than the M_2_AChR-immunized WT mice. This result indicates that the lack of P2×7 receptors impairs at least one important step in the chain of events, that includes antigen capture and processing by antigen-presenting cells, efficient antigen presentation to T cells and stimulation of M_2_AChR-specific B lymphocyte clones. One possibility is the need of functional expression of P2×7 receptors for efficient antigen processing and presentation by dendritic cells, as previously proposed by others^26^,^37^.

Despite the differences in the M_2_AChR antibody production between WT and P2×7^−/−^ mice, M_2_AChR P2×7^−/−^ animals also developed signs of DCM when compared with their pcDNA3 controls, such as a reduction in exercise time and an increase in the lumen of the left ventricle at the 40th week after the first immunization scheme.

The development of functional and morphological characteristics of DCM shown by the M_2_AChR P2×7^−/−^ mice surprised us since the importance of autoantibodies in the development of autoimmune DCM has been firmly established in the literature^38^. Besides this, a recent work showed that the treatment with the P2×7 receptor antagonist A740003 leads to an improvement in heart function of BALB/c male mice that develop autoimmune myocarditis after immunization with α myosin heavy chain^29^.

One possible explanation for the development of DCM by P2×7^−/−^ M_2_AChR-immunized mice would be a predisposition for the development of heart disease as these mice displayed a different electrocardiographic pattern to that observed for WT mice. To our knowledge this has not been previously reported in the literature. We described that P2×7^−/−^ animals presented a higher heart rate, longer QTc interval and superior ST depression than the WT mice.

The higher heart rate can be the result of the lack of P2×7 receptor at the sinoatrial node and/or in the nervous system. But its role in pacemaker activity has not been studied^39^,^40^. In addition, ATP and adenosine have negative chronotropic and dromotropic effects mediated by complex neuronal pathways involving P2X and P1 receptors and the binding of ATP to purinergic receptor that initiates a cascade facilitating the development of arrhythmias^29^,^41^.

In all experiments, unimmunized and pcDNA3-immunized P2×7^−/−^ mice spontaneously displayed higher ST depression in their ECG. This depression was not dependent on the presence of anti-M_2_AChR antibodies. Although few articles describe this electrocardiographic finding in mice associated with signs of cardiac ischemia, in humans it is commonly related to hypokalemia^42^. Transgenic mice overexpressing erythropoietin (Epo-TG6 mice) are highly intolerant to exercise despite showing an increase in myocardial contraction force and having a ST depression after injection of norepinephrine, a ST profile that arises spontaneously in the P2×7^−/−^ mice. This change preceded acute heart failure, demonstrating a barrier between myocardial oxygen demand and delivery^43^. We thus conclude that together with the increased heart frequency, the presence of ST depression suggests that P2×7^−/−^ mice have a predisposition to develop heart dysfunction.

Although the role of the P2×7 receptor in the heart is not completely clear yet, our data confirm its importance for the proper functioning of this organ, mainly regarding the electrical rhythm and conduction system. The cardiac differences between WT and P2×7^−/−^ mice led to the emergence of new issues that need further studies: (1) Are there any correlations between the development of heart disease in humans and gain or loss of function by P2×7 receptor? (2) Do patients with heart disease develop long-term changes in P2×7 receptor functionality? The answer to these questions would help to prevent and treat some cases of cardiac disease.

The transfer of anti-M_2_AChR antibodies from previously immunized M_2_AChR WT mice to P2×7^−/−^ naïve mice, as well as the transfer of serum from M_2_AChR P2×7^−/−^ mice that developed DCM, but did not display high titers of anti- M_2_AChR antibodies, did not induce the development of DCM in either WT or P2×7^−/−^ mice. These results suggest that other M_2_AChR-specific immune-related parameters, most likely a cellular immune response, may be important in the development of DCM in immunized P2×7^−/−^ mice. The possibility that this cellular component is also present in WT mice should also be considered.

We therefore analyzed other parameters of the immune response during the 40th week experimental period. We first analyzed Treg cell percentages in the spleens of WT and P2×7^−/−^ immunized mice. It is known that the presence of FoxP3^+^ cells protect against the development of autoimmune myocarditis in a different model^44^. In addition, the activation of the purinergic P2×7 receptor by ATP triggers a pro-inflammatory cascade, which induces pathways of regulatory T-cell death^45^. Accordingly, here we showed that P2×7 deficiency in C57BL/6J mice led to an increase in the percentage of FoxP3^+^CD4^+^ cells in the spleen compared to the WT group. A more detailed analysis of the IL-2 receptor alpha-chain (CD25) expression in the FoxP3^+^CD4^+^ sub-population demonstrated that P2×7^−/−^ mice displayed a significantly decreased percentage of CD4^+^CD25^+^FoxP3^+^ cells and significantly higher percentage of FoxP3^+^CD25^−^ cells. Although the origin and function of CD4^+^FoxP3^+^CD25^−^ cells is still quite controversial, it is believed that such cells would be more unstable with a higher tendency to lose the expression of FoxP3 and differentiate into effector T cells^35^,^46^,^47^,^48^. Thus, although we found no differences in Treg cell frequency between WT M_2_AChR and WT pcDNA3 groups, the P2×7^−/−^ pcDNA3 and M_2_AChR groups display a significant higher percentage of Tregs (compared to their respective WT group), due to higher frequency the unstable FoxP3^+^CD25^−^Treg sub-population, suggesting a decreased suppressor Treg cell activity or a higher conversion to effector cells in the M_2_AChR -immunized P2×7^−/−^ mice. Notably, CD4^+^Foxp3^+^CD25^−^ Tregs were found in type 1 diabetes mellitus^49^ and in systemic lupus erythematosus patients and some evidence suggest that these are dysfunctional Tregs^46^. However, FoxP3 expression in humans and mice differs in some aspects, as activated human T cells transiently express Foxp3 without the acquisition of suppressor potential. Therefore, further studies are needed to better clarify the identity and role of CD4^+^Foxp3^+^CD25^−^ cell population in autoimmunity.

The production of IL-17 at the 20^th^ week post-immunization increased in the M_2_AChR-immunized WT heart but not in the P2×7^−/−^ hearts, being reduced over a longer measured time. This increase could be one of the factors that contribute to the development of the disease in wild type mice as its influence has been shown in pro-fibrotic processes, as well as cardiac remodeling. After the establishment of dilatation the levels of IL-17 decrease, appearing to be essential to install a more severe disease feature^14^,^15^.

A study conducted with mononuclear cells isolated from patients infected with *T. cruzi* revealed that patients with moderate cardiomyopathy showed the highest levels of this cytokine, while patients classified as severe showed reduced levels of IL-17^21^. Moreover, it was demonstrated in mice with chronic dilated cardiomyopathy induced by *T. cruzi* infection that reduction of IL-17 levels, through the administration of anti-IL17, worsened the symptoms of the disease^20^. Therefore the reduction of IL-17 levels, or even the absence of its receptor-mediated signaling, could lead to a serious and fatal cardiomyopathy in mice^50^. However, the role of IL-17 appears to be strictly dependent on the context of other cytokines^16^.

We showed that IL-1 β followed a similar production pattern as the one presented by IL-17. At the 20^th^ week post-immunization the production of IL-1β was higher in M_2_AChR WT but not in the M_2_AChR P2×7^−/−^. A defect in the production of IL1-β in P2×7^−/−^ mice has been described in several experimental models^23^,^27^. In recent years, it has been demonstrated that IL-1β can stimulate the production of IL-17 in memory and naïve CD4^+^ T-cells. In addition, it has been shown that the pathway activated through IL-1R1 is necessary for the early differentiation of CD4^+^ Th17^51^ and that IL-1β and IL-23 induce the production of IL-17 in γδ cells. Moreover, γδ cells generate a loop amplification with CD4^+^ cells through an increase in the secretion of IL-17 in CD4^+^ cells^52^. In conclusion, the absence of ATP signaling in P2×7 receptor knockout mice provides a signaling framework to support the small production of IL17 and IL-1β.

The production of IFNgγ increased in the M_2_AChR WT and P2×7^−/−^ hearts at the 20^th^ week post-immunization, and, contrary to IL-1β, this effect was more remarkable in the P2×7^−/^ mice. High IFNγ levels have been described in several models of heart disease, mainly in Chagas’ disease^19^,^53^. IFNγ may increase the infiltration of inflammatory cells and the secretion of other pro-inflammatory cytokines leading to a significant down regulation of α-smooth muscle actin, disarraying the healing process, inducing myosin heavy chain degradation and cardiac remodeling^54^,^55^. High serum levels of IFNγ in overexpressing transgenic mice lead to cardiac dysfunction which culminates in the development of cardiomyopathy^56^. But in models of experimental autoimmune myocarditis, mainly induced by anti-myosin antibodies, IFNγ played a cardioprotective role, where mice that were deficient in IFNγ (*Ifn*γ^−/−^) developed a severe cardiomyopathy^16^,^57^.

Significant cardiac abnormalities were observed in M_2_AChR WT and M_2_AChR P2X^−/−^ mice that had peak production of IFNγ at the 20^th^ week post-immunization. These results highlight that the mechanisms which WT and P2×7^−/−^ mice immunized with pcDNA3-hM2 plasmid develop cardiomyopathy are through different pathways. One involves anti M_2_AChR antibodies and higher IL1β and IL17 cytokine levels, absent in the P2×7^−/−^ mice, and the other requires higher levels of IFNγ. M_2_AChR WT mice appear to be more dependent on the presence of anti-M_2_AChR antibodies, while M_2_AChR P2×7^−/−^ mice are more dependent on the mechanisms involving IFNγ described above. The involvement of IFNγ may be indicative of a T cell-mediated anti-M_2_AChR phenomenon. More experiments are needed to clarify this mechanism. In summary, our results confirm that the presence of anti-M_2_AChR antibodies lead to the development of DCM in WT mice. In addition, P2×7^−/−^ mice, which contain low titers of such antibodies, can also develop the disease, but in this case a cell-mediated immune response may become more prevalent. We also confirmed that IL-17 levels are inversely correlated with the severity of disease after establishment of the disease, which occurs primarily during the first 20 weeks post-immunization. This cytokine could participate in the cardiac remodeling process that triggers the DCM. In its absence, as was observed in the knockout mice, other factors (such as electrical pre-disposition) and cytokines (IFNγ) become more dominant factors in the development of the disease. Therefore, a precise balance of the immune response where a complex network of factors has been generated creates the conditions for the development of DCM.

## Methods

### Study design

Seven-week-old female C57BL/6J wild type (from Multidisciplinary Center for Biological Research - CEMIB, Campinas, São Paulo, Brazil) and Purinergic P2×7 receptor knockout mice (P2×7^−/−^) (from Jackson Laboratories – JAX® Mice and Services, Bar Harbor, Maine 04609, USA), weighing 16.0 g ± 0.7 g were randomly allocated to one of the following treatment groups: (1) wild type (WT) immunized with pcDNA3 plasmid (WT pcDNA3 group), (2) WT immunized with pcDNA3-hM_2_ (WT M_2_AChR group), (3) P2×7^−/−^, immunized with pcDNA3 plasmid (P2×7^−/−^ pCDNA3 group) and (4) P2×7^−/−^, immunized with pcDNA3-hM_2_ plasmid (P2×7^−/−^ M_2_AChR group) (n = 10 in each group). The animals from each group were individually identified and maintained at the local animal facility (Laboratory of Transgenic Animals - LAT, Institute of Biophysics Carlos Chagas Filho - IBCCF, UFRJ, Rio de Janeiro, Brazil). This study was approved by the Ethics Committee on the Use of Animals of Health Sciences Center of Federal University of Rio de Janeiro, Brazil (CEUA/CCS/UFRJ, CONCEA registered #01200.001568/2013.87, approved protocol IBCCF 041). All animals received humane care in compliance with the “Principles of Laboratory Animal Care” formulated by the National Society for Medical Research and the “Guide for the Care and Use of Laboratory Animals” prepared by the National Academy of Sciences, USA, and National Council for Controlling Animal Experimentation, Ministry of Science, Technology and Innovation (CONCEA/MCTI), Brazil.

The experimental mice, either native or P2×7KO, used in the present work were genotyped prior each experiment. For this, the genomic DNA from mice’ tail were extracted and submitted to PCR reaction using specific set of primers (native for wild type and neomycin for P2×7^−/−^). DNA samples corresponding to each animal was subjected to two PCR reactions, one with primers to amplify a fragment corresponding to the P2×7 native sequence and one with primers for amplification of a region containing part of the P2×7 neomycin chimera sequence, obtained by the replacement of the region of the gene encoding Cys506 to Pro532 with the neomycin resistance gene^58^. The amplification of a unique fragment of 418 bp corresponding to the native sequence was observed only when genomic DNA from wild type was used as template. None of the samples of genomic DNA originated from P2×7^−/−^ mice showed PCR product amplification using these native primers. Contrarily, these latter samples showed amplification bands of 510 bp at agarose gels, only in reactions were the 3´neomycin primer was used for amplification) confirming that P2×7^−/−^ animals carry the mutant allele.

The immunizations were performed using a helium-driven gene gun (Bio-Rad, Hercules, CA, USA) as previously described^22^. Each mouse was primed and boosted two more times, with a 14 day interval between boosts (days 0, 14 and 21). The immunization scheme was repeated starting at the 20^th^ week after the first immunization scheme. All procedures performed with these mice are described in detail in subsequent sessions and summarized in Supplementary Figure S3.

### Sera collection and enzyme-linked immunosorbent assays (ELISA)

Blood samples were collected from the mouse orbital sinus under anesthesia with ketamine 60 mg/Kg and xilazine 3 mg/Kg at a mean interval of 4 weeks over the course of 40 weeks (immune samples), including samples prior to the first immunizations (pre-immune samples). For serum separation the samples were maintained at 25 °C, overnight, centrifuged twice at 5.000 × *g* for 15 minutes and the clear serum layer was stored in approximately 200 μL at −80 °C. The quantification of IgG antibody titers was performed by enzyme-linked immunosorbent assay (ELISA) using a synthetic peptide corresponding to M_2_AChR-el_2_ (_168_VRTVEDGECYIQFFSNAAVTFGTAI_192_) as previously described^22^.

### Cardiac exercise testing analysis

The animals were subjected to ergometric tests before immunization (seven weeks old) and at the 35^th^ week after the first immunization using a treadmill (Panlab, Cornella, Barcelona) with an adjustable speed and a 10° slope. The treadmill was equipped with electrodes, producing shocks of 0.5 mA activated when the animal tried to rest. Two days before the functional test, the mice were subjected to room recognition for 15 minutes at 17 cm/s. On the functional test day, the mice were placed in the treadmill chamber at a speed of 17 cm/s. Every 2 minutes, the speed was increased by 2 cm/s. The animals ran until exhaustion when the total time achieved was recorded.

### Morphometric analysis

The pcDNA3 and M_2_AChR mice were euthanized through cervical dislocation preceded by anesthesia with ketamine 60 mg/Kg and xilazine 3 mg/Kg. The heart of each animal was removed from base vessels while still mechanically active and immediately immersed in PBS (Phosphate-buffered saline). The apex and the base of each heart were removed and the remaining tissue was photographed. To quantify left ventricular dilation, the internal area and the total area of left ventricle cross section were measured using ImageJ software (version 1.45s, the National Institutes Health, USA). Subsequently, the relationship between these two measurements was plotted using GraphPad Prism (version 5 for Windows, GraphPad Software, San Diego California USA, www.graphpad.com).

### Electrocardiographic analysis

Electrocardiographic (ECG) evaluation was performed at the 5^th^, 10^th^, 20^th^ and 40^th^ weeks post-immunization. All animals were anaesthetized through intraperitoneal injection of ketamine 60 mg/Kg and xilazine 3 mg/Kg. After sedation, the animals were placed on a platform in the prone position. ECG was recorded by connecting the four limbs to a Bio Amp PowerLab 2/20 System (AD Instruments, Castle Hill, Australia), using the bipolar lead I configuration. The digitalized ECG signals were recorded and stored for posterior analysis using the LabChart 6 program (AD Instruments). The ECG analysis included the following measurements: heart rate, PR interval, P wave duration, RR interval, QT interval, QTc, QRS complex duration, ST height, JT interval and arrhythmia. The ECG recordings were obtained from each animal for 15 minutes and all recordings were performed in the morning period.

### Serum Transfer

The serum collected from pcDNA3 and M_2_AChR WT and P2×7^−/−^ immunized mice at the 5^th^ and 10^th^ weeks post-immunization, previously described above, were pooled and stored at −20 °C until use. 8-week-old C57BL/6J female recipient mice (N = 5) received 100 μL of serum pool intravenously according to a regime of three transfers at intervals of 14 days. After 10 weeks post-transfer the recipient mice were subjected to ECG following the same specifications described before.

### Regulatory T cell analysis

Treg cell analyses were performed by flow cytometry using a FACSCalibur flow cytometer (BD Biosciences, San Jose, CA, USA). The spleens were removed aseptically and mechanically dissociated in the presence of DMEM medium. The dissociated cells were centrifuged and the pellet was resuspended in hemolytic solution, followed by additional centrifugation. The pellet was resuspended in DMEM medium supplemented with 5% fetal bovine serum and the numbers of splenocytes were counted using a Neubauer chamber. Erythrocyte-depleted spleen cells (2 × 10^6^ cells) were incubated with anti-CD4-FITC (BD Pharmingen) and anti-CD25-APC (BD Pharmingen) at 4 °C for 30 minutes, followed by washing with FACS Buffer (PBS, 0.5% BSA and 0.01% NaN_3_). After this, cells were fixed and permeabilized using the Mouse Regulatory T-Cell Staining Kit (eBioscience) and stained with anti-mFoxP3-PE (eBiosciences), according to the manufacturer’s instructions. The data were analyzed using cellquest™ Pro Software (BD Biosciences).

### Cytokine quantification using ELISA

IL-17 (Mouse IL-17 ELISA, Bender Med System, Vienna, Austria), IFNγ (Murine IFN-γ Mini ELISA Development Kit, PepoTrech, Rocky Hill, NJ, United States) and IL-1β (Mouse IL-1β ELISA Set, BD Bioscience, Franklin Lakes, NJ, USA) expression in the heart were analyzed using specific ELISA kits. All procedures were performed following the manufacturers’ recommendations.

Heart tissue fragments (approximately 100 mg) were homogenized using an Ultra-Turrax T25 basic homogenizer (IKA Werke GmbH & Co. KG, Staufen, Germany) in cold lysis buffer (50 mM HEPES, 1 mM MgCl_2_ and 10 mM EDTA, pH 7.4 on ice). The macerated tissue was centrifuged (15.000 × *g* for 15 minutes at 4 °C) and the supernatants were collected for cytokine quantification. All assays were performed in triplicate.

### Statistical analysis

The statistical significance was evaluated using two-way analysis of variance (ANOVA) with Bonferroni’s multiple comparisons test for the comparison between groups. The differences between parameters obtained at pre-immunization compared with those obtained at 5, 10, 20 and 40th weeks post-immunization are marked by asterisks (*p < 0.05; **p < 0.01; and ***p < 0.001). Comparisons between WT pcDNA3 and WT M_2_AChR and between P2×7^−/−^ pcDNA3 and P2×7^−/−^ M_2_AChR were marked with the letters **a** and **b**, respectively. Comparisons between WT pcDNA3 and P2×7^−/−^ pcDNA3 and between WT M2AChR and P2×7^−/−^ M2AChR were marked by the letter **c** and **d**, respectively. Statically significant differences between WT and P2×7^−/−^ before immunization (0 weeks post-immunization) were marked by letter e. In these cases p < 0.05 was considered statistically significant. These analyses were performed using GraphPad Prism 5.00 personal computer software. The graphs were plotted using the means ± S.D, except for anti-M_2_AChR-el_2_ titer quantification that was represented by the means ± S.E.M.

## Additional Information

**How to cite this article**: Martinez, C. G. *et al.* P2 × 7 purinerginc signaling in dilated cardiomyopathy induced by auto-immunity against muscarinic M_2_ receptors: autoantibody levels, heart functionality and cytokine expression. *Sci. Rep.* **5**, 16940; doi: 10.1038/srep16940 (2015).

## Supplementary Material

Supplementary Materials

## Figures and Tables

**Table 1:** Electrocardiogram recordings in Wild Type (WT) and P2×7^−/−^ immunized mice.

Wild Type									
	0 wks p.i	5 wks p.i		10 wks p.i		20 wks p.i		40 wks p.i	
		pcDNA3	M_2_AChR	pcDNA3	M_2_AChR	pcDNA3	M_2_AChR	pcDNA3	M_2_AChR
RR interval (ms)	246.47 ± 22.78	225.33 ± 14.23	260.94 ± 42.24	262.98 ± 11.97	220.70 ± 13.65	175.78 ± 30.50	181.16 ± 56.15	268.85 ± 38.12	250.80 ± 18.90
Heart Rate (BPM)	248.11 ± 34.94	245.33 ± 43.20	232.30 ± 35.42	222.63 ± 13.17	251.96 ± 40.91	306.80 ± 65.03	342.26 ± 95.11	213.77 ± 24.17	229.53 ± 43.39
QRS interval (ms)	10.35 ± 2.26	11.22 ± 0.86	9.46 ± 1.07	10.38 ± 0.95	9.07 ± 0.59	11.07 ± 0.62	10.56 ± 1.09	11.03 ± 1.42	11.31 ± 1.13
QTc interval (ms)	44.00 ± 4.29	39.39 ± 6.30	40.60 ± 5.12	44.24 ± 1.85	45.23 ± 4.17	57.80 ± 12.86	56.68 ± 13.24	63.66 ± 18.29	57.05 ± 5.47
ST Height (mV)	0.15 ± 0.07	0.09 ± 0.07	0.11 ± 0.01	0.08 ± 0.01	0.19 ± 0.04 ^**a**^	0.06 ± 0.04	0.10 ± 0.02	0.13 ± 0.04	0.10 ± 0.05
JT interval (ms)	11.41 ± 2.08	11.89 ± 6.25	11.12 ± 1.58	12.30 ± 1.80	12.79 ± 1.49	14.79 ± 1.24	12.27 ± 3.48	18.47 ± 5.50	17.44 ± 1.92
P2×7^−/−^									
RR interval (ms)	189.63 ± 16.24^**e**^	192.15 ± 16.38	208.20 ± 16.36^**d**^	193.43 ± 51.37^**c**^	241.83 ± 25.87^**b**^	152.52 ± 31.71	182.00 ± 14.35	244.06 ± 17.43	239.48 ± 10.43
Heart Rate (BPM)	311.19 ± 30.14^**e**^	282.78 ± 73.05	287.18 ± 20.29	353.47 ± 32.13^**c**^	235.90 ± 21.21^**b**^	390.06 ± 85.31	303.83 ± 13.45	274.57 ± 31.17	252.97 ± 5.84
QRS interval (ms)	8.88 ± 2.03	11.64 ± 3.05	9.17 ± 2.19	9.81 ± 1.37	10.73 ± 3.40	9.98 ± 1.51	10.43 ± 2.07	10.05 ± 2.39	12.89 ± 2.52
QTc interval (ms)	48.80 ± 5.05	52.58 ± 11.01	42.87 ± 4.61	61.00 ± 18.85 ^c^	56.19 ± 6.87	63.00 ± 2.79	61.40 ± 10.34	58.20 ± 8.26	57.02 ± 6.16
ST Height (mV)	0.06 ± 0.06^**e**^	0.00 ± 0.08	−0.03 ± 0.08^**d**^	−0.01 ± 0.04	−0.02 ± 0.10^**d**^	−0.01 ± 0.09	−0.07 ± 0.03^**d**^	−0.01 ± 0.09^**c**^	−0.01 ± 0.11
JT interval (ms)	11.18 ± 3.11	11.51 ± 6.98	9.43 ± 1.28	14.33 ± 6.04	15.11 ± 4.32	15.34 ± 1.69	13.04 ± 3.90	18.23 ± 4.26	15.00 ± 2.50

Comparison between WT pcDNA3 and WT M_2_AChR and between P2×7^−/−^ pcDNA3 and P2×7^−/−^ M_2_AChR are marked with the letters a and b, respectively. Comparison between WT pcDNA3 and P2×7^−/−^ pcDNA3 and between WT M_2_AChR and P2×7^−/−^ M_2_AChR are marked by the letter c and d, respectively. Comparison between WT and P2×7^−/−^ previously immunization scheme was marked by letter e. p < 0.05 was considered statistically significant difference. N = 10. Wks p.i. – Weeks post-immunization.

**Table 2:** Electrocardiogram recordings in mice that received pcDNA3 or M_2_AChR WT serum 10 weeks post-serum transfer.

	Wild Type Recipient	P2×7^−/−^Recipient	pcDNA3 Wild type serum	M_2_AChR Wild type serum
	pcDNA3 Wild type serum	M_2_AChR Wild type serum		
RR Interval (ms)	210.45 ± 45.60	138.97 ± 10.53^**a**^	147.70 ± 21.24^**c**^	168.77 ± 27.33
Heart Rate (BPM)	337.20 ± 81.22	379.23 ± 13.70	384.23 ± 52.29^**c**^	359.15 ± 55.39
PR Interval (ms)	40.44 ± 8.55	34.84 ± 8.68	39.13 ± 1.48	40.10 ± 0.45
QRS Interval (ms)	9.63 ± 1.37	13.21 ± 1.25	13.86 ± 1.97	13.86 ± 1.52
QTc Interval (ms)	66.68 ± 1.13	85.43 ± 2.08	83.39 ± 2.91	74.89 ± 2.52
ST Height (mV)	0.020 ± 0.054	0.115 ± 0.057	−0.050 ± 0.022	- 0.015 ± 0.070^**d**^
JT interval	12.91 ± 1.14	19.07 ± 0.76^**a**^	16.88 ± 6.85	12.24 ± 2.92^**d**^

Comparison between WT pcDNA3 and WT M_2_AChR and between P2×7^−/−^ pcDNA3 and P2×7^−/−^ M_2_AChR are marked with the letters a and b, respectively. Comparison between WT pcDNA3 and P2×7^−/−^ pcDNA3 and between WT M_2_AChR and P2×7^−/−^ M_2_AChR are marked by the letter c and d, respectively. p < 0.05 was considered statistically significant difference. N = 5.
