# Supplementary material for: P2×7 purinergic signaling in dilated cardiomyopathy induced by auto-immunity against muscarinic M2 receptors: autoantibody levels, heart functionality and cytokine expression
Source: Sci Rep. 2015 Nov 23;5:16940. doi: 10.1038/srep16940 (PMC4655336; doi:10.1038/srep16940)
Supplement: Supplementary Materials [file srep16940-s1.pdf]

P2X7 purinerginc signaling in dilated cardiomyopathy induced by auto-immunity against muscarinic M_2 receptors: autoantibody levels, heart functionality and cytokine expression

Camila Guerra Martinez[1,4], Daniel Zamith-Miranda[2], Marcia Gracindo da Silva[1,4], Karla Consort Ribeiro[5],Izaíra Trincani Brandão[3], Celio Lopes Silva[3], Bruno Lourenço Diaz[1], Maria Bellio[2], Pedro Muanis Persechini[1,4], and Eleonora Kurtenbach*[1,4]

(1) Instituto de Biofísica Carlos Chagas Filho, Universidade Federal do Rio de Janeiro, Rio de Janeiro, RJ, 21941-902, Brasil.
(2) Instituto de Microbiologia Prof. Paulo de Goes, Universidade Federal do Rio de Janeiro, 21941-900 Rio de Janeiro, RJ, Brasil.
(3) Departamento de Bioquímica e Imunologia, Faculdade de Medicina de Ribeirão Preto, Universidade de São Paulo, Av. Bandeirantes 3900, Ribeirão Preto, SP, Brasil.
(4) Instituto Nacional de Ciência e Tecnologia para Pesquisa Translacional em Saúde e Ambiente na Região Amazônica, Conselho Nacional de Desenvolvimento Científico e Tecnológico/MCT, Rio de Janeiro, Brasil.
(5) Instituto Nacional de Propriedade Industrial. Rua São Bento no 1, Rio de Janeiro, RJ, 20090-010, Brazil.

* Corresponding author: Eleonora Kurtenbach, Instituto de Biofísica Carlos Chagas Filho, Centro de Ciências da Saúde, Universidade Federal do Rio de Janeiro, Av. Carlos Chagas Filho 343, Cidade Universitária, 21941–902, Rio de Janeiro, RJ, Brazil. E-mail: kurten@biof.ufrj.br. Phone: 55-21-39386573, Fax: 55-21-22708647.

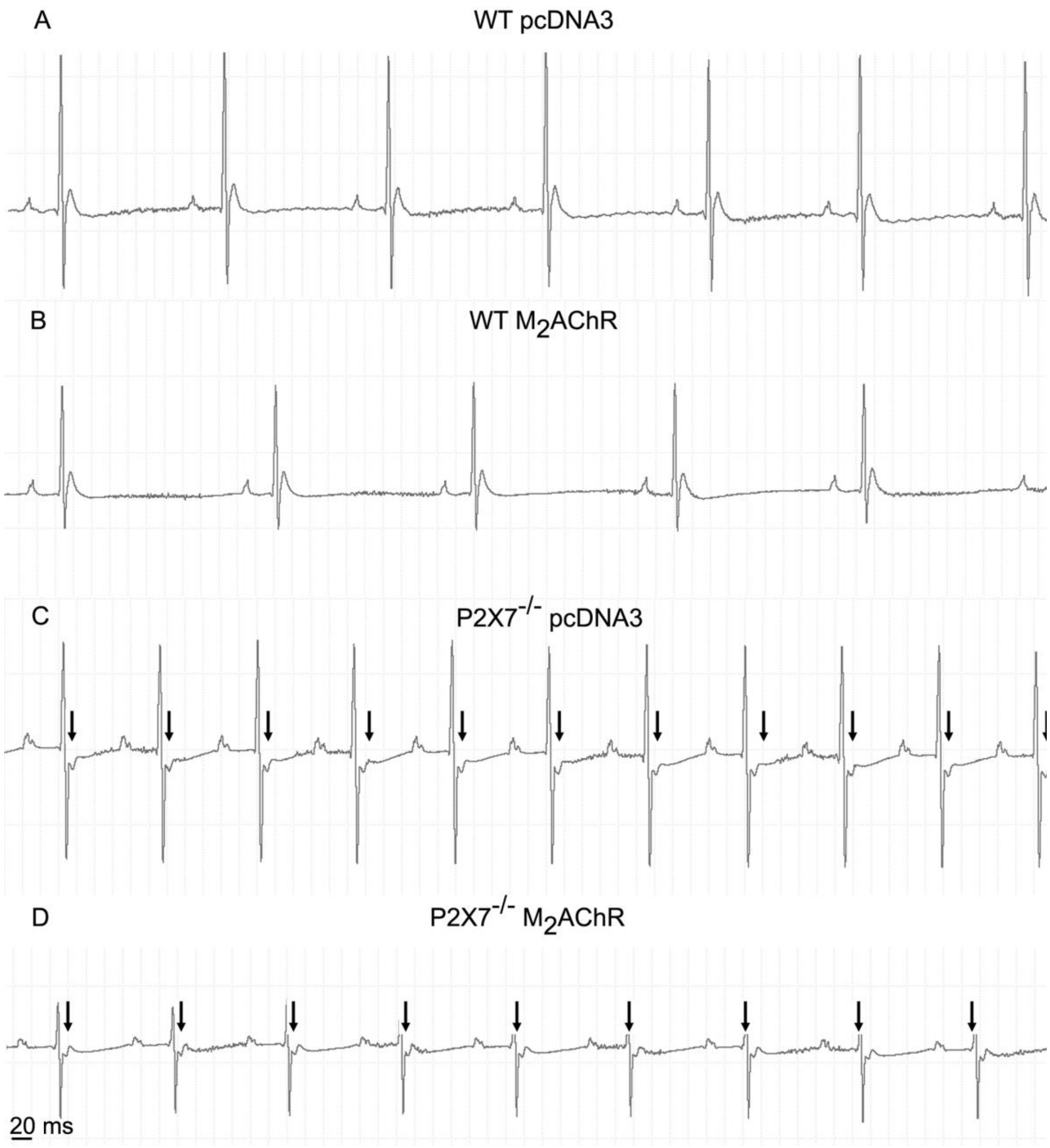

Supplementary figure S1 – **Representative electrocardiogram (ECG) recordings in WT and P2X7$^{-/-}$ immunized animals at 20th weeks post-immunization.** The animals were anesthetized with ketamine and xylazine prior to the exam. All ECGs were performed in the DI lead. The results obtained from all exams are described in Tables 1 and 2. These are the first published ECG recordings in P2X7$^{-/-}$ mice that we have knowledge after extensive review of the literature. P2X7$^{-/-}$ mice, belonged to pcDNA3 and M_2AChR groups, presented segment ST depressions (arrows) that were not observed in any WT mice.

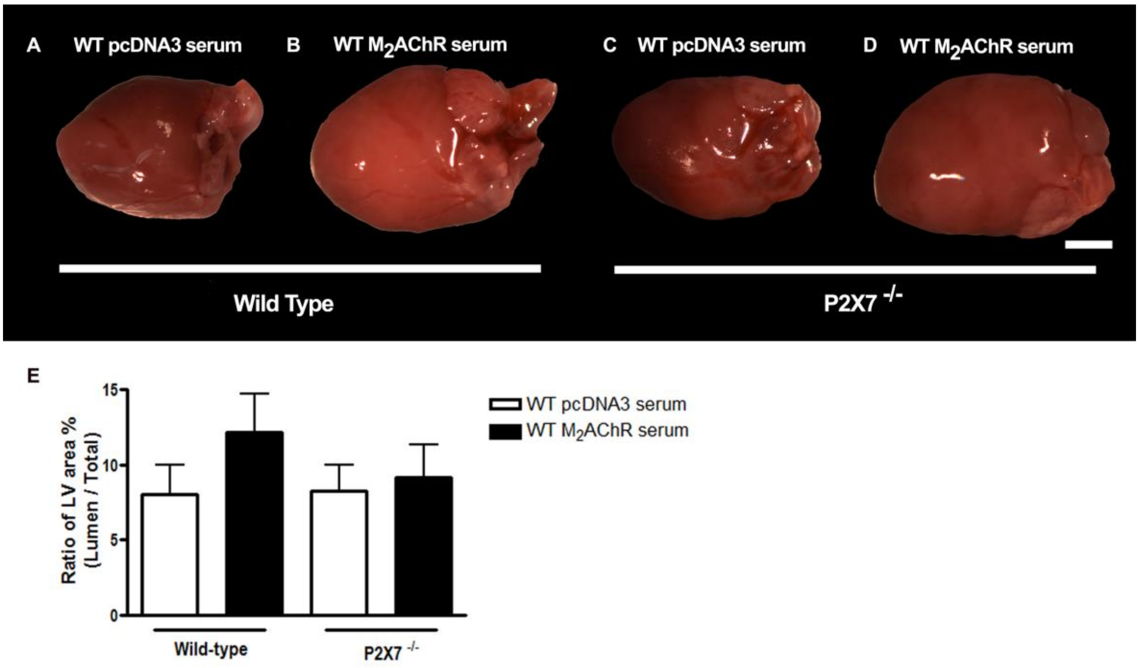

Supplementary figure S2 - **Morphological assessment of cardiac dilatation at 10th week post-serum transfer.** Hearts' photographs of wild type (WT – A and B) and P2X7$^{-/-}$ mice (C and D) that received pcDNA3 WT serum (A and C) or M_2AChR WT serum (B and D). Scale bar, 1 mm. (E) Cardiac tissue was fixed in 4% formaldehyde (Polysciences), mounted in historesin (Leica) and sections of 1 μm were obtained on a microtome (Leica RM 2155). These sections were stained with hematoxylin and eosin and analyzed by Stereomicroscope (Nikon SMZ 800) coupled with an image capture system to study tissue morphology. For quantification, the area of left ventricle, lumen and total, was measured by Image Pro-Plus software and the ratio between these two measures was plotted using GraphPad Prism ®

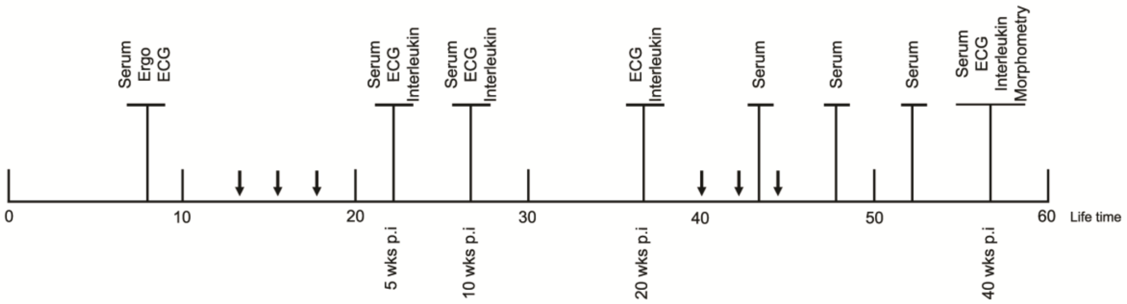

Supplementary figure S3 - **Study design.** The life time of the mice are shown in weeks since the date of birth for 57 weeks (shown horizontally). The small vertical bars indicate an interval of 10 weeks. Two sets of immunization were performed (arrows); the first one started at the 13 week after birth and the 2nd one started at the 40th week after birth (23 weeks after the end of the first immunization scheme). The immunization (arrows) occurred in three steps with an interval of 15 days between them. Cardiac function and immunological parameters were assessed over time in the weeks marked with larger vertical bars. ECG - Electrocardiogram. Ergo – Ergometry. Wks p.i. – Weeks post-immunization. Interleukin – Heart interleukin dosage. Serum - Serum processing for anti-M_2AChR-el_2 titration analysis.

Supplementary Table S1 - Representative photographs of mice hearts (upper panel) and ECG data (lower panel) from wild type and P2X7$^{-/-}$ recipients mice of P2X7$^{-/-}$-immunized serum at 10 weeks post-serum transfer. Comparisons between WT pcDNA3 and WT M_2AChR and between P2X7$^{-/-}$ pcDNA3 and P2X7$^{-/-}$ M_2AChR were marked with the letters **a** and **b**, respectively. Comparisons between WT pcDNA3 and P2X7$^{-/-}$ pcDNA3 and between WT M_2AChR and P2X7$^{-/-}$ M_2AChR were marked by the letter **c** and **d**, respectively. Comparison between WT and P2X7$^{-/-}$ previously immunization scheme was marked by letter **e**. $p < 0.05$ was considered statistically significant. N = 10. Scale bar = 1 mm

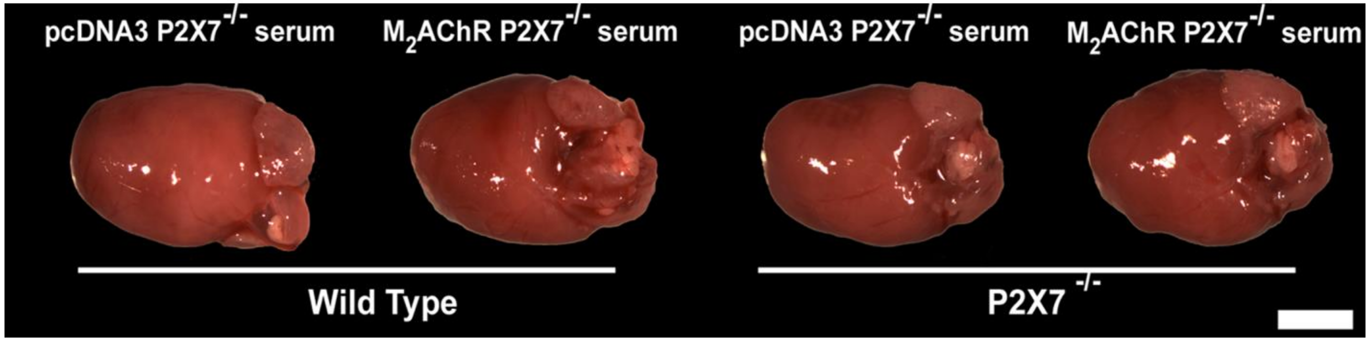

	Wild Type Recipient		P2X7$^{-/-}$ Recipient	
P2X7$^{-/-}$ immunized serum	pcDNA3	M_2AChR	pcDNA3	M_2AChR
RR Interval (ms)	228.63 ± 11.35	178.87 ± 54.24	164.10 ± 29.59 [c]	156.70 ± 23.53

Heart Rate (BPM)	342.30 ± 12.85	332.40 ± 102.68	386.40 ± 63.93 [c]	374.27 ± 73.93
PR Interval (ms)	43.83 ± 6.03	43.02 ± 4.98	45.27 ± 2.28	46.04 ± 1.32
QRS Interval (ms)	12.89 ± 0.81	12.39 ± 2.97	16.08 ± 2.44	10.98 ± 4.28
QTc Interval (ms)	63.98 ± 10.01	60.80 ± 14.23	63.61 ± 11.25	63.24 ± 5.45
ST Height (mV)	0.04 ± 0.03	0.09 ± 0.06	- 0.04 ± 0.04 [c]	- 0.05 ± 0.03 [d]
JT interval	14.45 ± 1.81	14.68 ± 3.45	8.69 ± 1.31 [c]	10.93 ± 3.49
